# The Ethical Cost of Delay: Lessons From an Acute Haemorrhagic Neurological Emergency in a Low‐Resource Setting

**DOI:** 10.1002/ccr3.73019

**Published:** 2026-06-25

**Authors:** Kwasi Asare Boadi, Dennis Awedam Achio, Eunice Wilberforce A. Achio

**Affiliations:** ^1^ Asunafo‐South District Hospital Ahafo Region Ghana; ^2^ University of Ghana Medical Centre Accra Ghana

**Keywords:** case report, emergency referral pathways, health system ethics, intracerebral hemorrhage, intraventricular hemorrhage, low‐resource setting, stroke care delays

## Abstract

Prompt recognition and early intervention are critical in the management of intracerebral hemorrhage. This case demonstrates how system‐level diagnostic and referral delays in a low‐resource setting can worsen outcomes, underscoring the ethical imperative for coordinated emergency pathways and equitable access to neurosurgical care.

## 1 Introduction

Stroke remains a major contributor to global morbidity and mortality, accounting for a significant proportion of neurological disability worldwide [1]. Intracerebral hemorrhage (ICH) is less common than ischemic stroke, comprising approximately 37.6% of all stroke cases globally, while ischemic strokes account for about 62.4% [2, 3]. In low‐ and middle‐income countries (LMICs), however, haemorrhagic strokes constitute a higher proportion of stroke presentations, with some studies reporting rates of up to 34% [4].

Despite their lower incidence, haemorrhagic strokes are associated with disproportionately high morbidity and mortality. Intraventricular extension of haemorrhage is particularly associated with poor outcomes due to complications such as obstructive hydrocephalus, raised intracranial pressure, and early neurological deterioration [5]. The successful management of acute stroke depends on rapid diagnosis, prompt neuroimaging, and timely initiation of appropriate care.

Paradoxically, LMICs where the burden of ICH is greatest are often the least equipped to provide effective acute stroke care. In Ghana and similar contexts, studies have demonstrated critical delays across the continuum of stroke management, including delays in accessing diagnostic imaging and specialist neurosurgical services [6]. Data from sub‐Saharan Africa indicate that the median time from symptom onset to neuroimaging often exceeds 6 h in district‐level facilities, substantially beyond the windows recommended in international guidelines [7, 8]. Another challenge is the fact that most LMICs do not meet the WHO quota of 1 neurologist per 100,000 persons [9]. Only Algeria, Egypt, and Tunisia have met or exceeded this quota [10] Available of neuroimaging modalities required for prompt diagnosis is another factor limiting effective management of strokes in LMICs. There is less than 1 CT scanner per million inhabitants in LMICs compared to almost 40 scanners per million inhabitants in high‐ income countries (HICs) [11]. Less than 15% of hospitals in Africa have dedicated stroke units where the best practices in stroke management are routinely administered [12]. These delays significantly increase the risk of unfavorable outcomes. The data described above show that the burden of stroke is increasingly shifting toward low‐ and middle‐income countries even as the infrastructure required to manage it remains concentrated in high‐income settings. This case is therefore particularly significant in the current global healthcare environment. Documenting and analyzing cases such as this one contributes to an evidence base that is essential for advocacy, policy reform, and the reorientation of health system investment toward emergency care infrastructure in resource‐limited settings.

Beyond clinical implications, such delays raise ethical concerns relating to justice, fairness, and equity in access to emergency care. They reflect systemic deficiencies that undermine the ethical foundations of health systems in resource‐limited settings.

We present the case of a 62‐year‐old man with acute intracerebral hemorrhage whose outcome was significantly influenced by system‐level diagnostic and referral delays, highlighting the intersection between emergency care delivery and medical ethics.

## 2 Case History/Examination

A 62‐year‐old retiree with a known history of poorly controlled hypertension presented following sudden loss of consciousness while playing draughts with friends. He was formerly a public servant who had no formal employment at the time of the incident. Collateral history revealed that he had complained of a sudden‐onset, severe headache shortly before requesting a ‘pain killer’. He subsequently slumped in his seat, developed facial deviation with drooping of the lips to the left, and became unresponsive. He was breathing spontaneously but was unarousable and was transported immediately to the Nima Government Clinic at 10:30 a.m.

On arrival, primary survey findings were as follows:

- Random blood sugar: 9.8 mmol/L.
- Airway: Patent; oxygen saturation 98% on room air.
- Breathing: Respiratory rate 16 cycles/min, regular; vesicular breath sounds bilaterally.
- Circulation: Blood pressure 210/109 mmHg; pulse 84 beats/min.
- Disability: Glasgow Coma Scale (GCS) score of 7/15 (E1, V2, M4); pupils sluggishly reactive to light. Focal neurological deficits could not be reliably assessed due to the reduced level of consciousness.

Intravenous labetalol 20 mg was administered slowly over 2 min for blood pressure control. Jaw thrust and chin‐lift maneuvers were performed to ensure airway patency and close neurological monitoring was initiated.

## 3 Differential Diagnosis, Investigations, and Treatment

- Subarachnoid hemorrhage
- Acute intracerebral hemorrhage
- Acute ischaemic stroke.

Basic laboratory investigations including complete blood count, hba1c, lipid profile, renal function test and coagulation profile were requested. A non‐contrast CT scan of the brain (Figure 1) was urgently requested to differentiate these conditions. The patient was transferred to an external imaging center for CT scanning, as the facility lacked on‐site neuroimaging capability. The scan was completed at approximately 1:15 pm, approximately 165 min after the initial presentation. Efforts to secure referral to a facility with neurosurgical capability were initiated in parallel.

**FIGURE 1:**
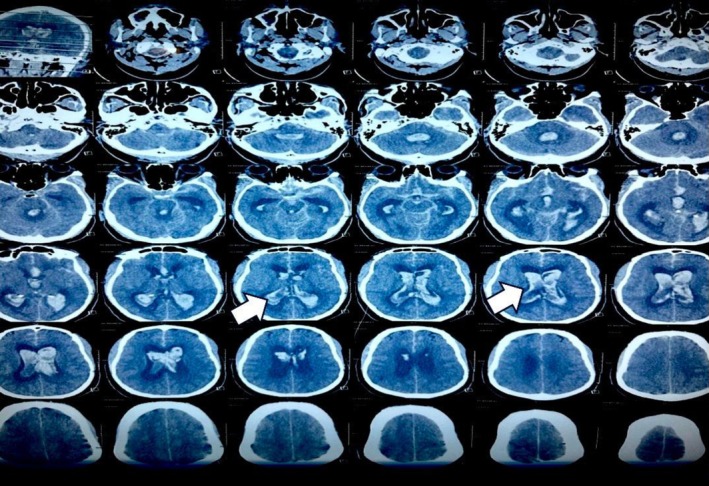
Non‐contrast CT scan. Arrows show extensive intraventricular hemorrhage involving the third, fourth, and lateral ventricles.

### 3.1 Imaging Findings

A non‐contrast CT scan of the brain revealed:

- Extensive acute intraventricular hemorrhage involving both lateral ventricles, the third, and fourth ventricles
- Moderate obstructive hydrocephalus with ventricular ballooning
- Subarachnoid hemorrhage involving the frontal lobes, bilateral Sylvian fissures, and basal cisterns. Diffuse cerebral oedema with loss of gray–white matter differentiation
- Posterior fossa crowding without definitive herniation
- No midline shift, parenchymal hematoma, or mass lesion

The coexistence of extensive intraventricular and subarachnoid hemorrhage without an identifiable parenchymal source was considered atypical. The basal ganglia, thalami, and cerebellum appeared preserved. These findings indicated a severe, high‐grade hemorrhage. While the absence of a discrete parenchymal clot reduced the immediate surgical target, the degree of intraventricular extension and hydrocephalus represented a potential indication for external ventricular drainage (EVD) at a tertiary centre.

Following return from the imaging centre, the patient experienced a generalized tonic seizure that self‐aborted within 20 s, without associated urinary or fecal incontinence.

The patient was managed with:

- IV dexamethasone 12 mg stat (to reduce vasogenic cerebral oedema)
- IV 20% mannitol 250 mL three times daily over 24 h (osmotic therapy to reduce intracranial pressure)
- Nursing in a 30‐degree head‐up position (standard positioning to facilitate venous drainage and reduce intracranial pressure)
- Vital signs monitoring every 30 min
- Strict input and output charting
- IV paracetamol 1 g three times daily
- Placement of a nasogastric tube and urethral catheter
- Seizure monitoring with a seizure chart

Repeated attempts to secure referral to tertiary facilities were unsuccessful. The first referral call was made at approximately 2:00 pm on Day 1, within 45 min of CT results being available. The response was that the Intensive Care Unit (ICU) was full. A private facility with intensive care and neurosurgical services required an upfront deposit of approximately GHC 50,000 prior to acceptance, which the family was unable to provide. Attempts to reach other public tertiary facilities were also unsuccessful due to reported bed unavailability. Supportive care continued while referral efforts persisted. Ultimately, supportive care is not a definitive treatment. The fact that definitive treatment was not initiated worsened the prognosis as processes related to secondary brain injury such as cerebral oedema, inflammation and release of oxidative species are known to set in following the primary brain injury from the haemorrhage.

## 4 Outcome and Follow‐Up

On Day 2, the patient's blood pressure was 190/95 mmHg, pulse 69 beats/min, oxygen saturation 98% on room air, random blood sugar was 7.9 mmol/L and temperature 36.4°C. Neurological examination showed further deterioration, with a GCS score of 4/15 (E1, V2, M1) and sluggish pupillary responses.

A bed was eventually secured at a tertiary facility, and the patient was transferred via ambulance at approximately 4:00 pm on Day 2, more than 24 h after the initial event. He died shortly after admission to the intensive care unit.

## 5 Discussion

This case highlights a recurrent challenge in the management of neurological emergencies in LMICs. Although the patient presented promptly to a healthcare facility, cumulative delays in diagnosis and referral significantly worsened his prognosis.

The absence of on‐site neuroimaging necessitated transfer to an external diagnostic centre, resulting in critical time loss. Clinical features alone are often insufficient to distinguish haemorrhagic from ischaemic stroke, making neuroimaging essential [13, 14]. Non‐contrast CT scanning remains the gold standard for the initial diagnosis of ICH, enabling assessment of haemorrhage extent, intraventricular involvement, mass effect, and raised intracranial pressure [15, 16].

Further delay occurred while attempting to secure referral to a facility with neurosurgical and intensive care capacity. Management of ICH in dedicated stroke units is associated with reduced mortality and improved functional outcomes [17]. Early neurological deterioration occurs in up to 23% of patients within the first hours of presentation [18], and prolonged emergency department stays and delayed ICU transfer are independently associated with worse outcomes [19].

It is important to acknowledge that intracerebral hemorrhage carries a high baseline mortality, approximately 40% at 30 days, even in well‐resourced settings with rapid neurosurgical access [20]. This epidemiological reality does not diminish the ethical concerns raised by this case but rather reframes them. The central argument is not that delay caused this patient's death with certainty. The argument is that systemic and financial barriers denied him the clinical assessment, specialist evaluation, and potential neurosurgical intervention, including possible external ventricular drainage for his obstructive hydrocephalus, that represent the standard of care to which any patient is entitled. The ethical violation lies in the deprivation of a chance, not the guarantee of a different outcome. The decision about whether this patient fell within the survivable majority was never made by clinicians. It was made by factors like geography, infrastructure, and financial capacity. This is the essence of distributive injustice in emergency medicine.

The requirement for substantial upfront payment prior to acceptance at a private facility underscores significant ethical concerns. The patient's outcome was influenced by financial capacity rather than clinical need, raising issues of distributive justice, beneficence, and non‐maleficence. The ethical principles implicated in this case exist in structural tension within resource‐limited settings. Distributive justice demands that emergency care be allocated based on clinical need. Yet when referral is contingent on financial capacity and neurosurgical services are inaccessible, the principle of beneficence becomes operationally constrained for the frontline clinician. Non‐maleficence is similarly compromised. The harm in this case arose not from an act of commission but from a systemic inability to act at all. Balancing these principles in low‐resource contexts therefore requires intervention at the health system level and not merely at the bedside. These principles which are rooted in both biomedical ethics and international human rights law demand that emergency care pathways be structured so that triage is governed by clinical urgency alone.

International guidelines recommend that emergency departments be equipped to manage ICH or have clearly defined pathways for rapid transfer to tertiary centres [21]. The facilities to effectively manage such conditions or the means to ensure prompt referral are absent in most district‐level facilities in Ghana and comparable settings. Their applicability in such contexts is therefore aspirational rather than operational.

## 6 Conclusion

Early recognition of intracerebral hemorrhage must be matched by rapid and coordinated system‐level responses. Outcomes in intraventricular hemorrhage depend not only on clinical expertise but also on health system efficiency. While the high baseline mortality of ICH means that outcomes cannot always be altered by faster care, the ethical obligation to provide equitable access to assessment and intervention remains unconditional. This case underscores the need for standardized referral protocols, strengthened emergency care pathways, and equitable access to neurosurgical services in resource‐limited settings.

## 7 Recommendations

Based on the findings of this case, we offer the following recommendations:

1. District and polyclinic facilities in Ghana and comparable LMICs should be prioritised for point‐of‐care neuroimaging capacity to eliminate diagnostic transfer delays in time‐sensitive neurological emergencies.
2. National emergency referral protocols should be formalised and enforced, with pre‐negotiated acceptance agreements between district facilities and tertiary centres that remove financial preconditions for emergency admission.
3. The Ghana Health Service and comparable ministries of health should audit delay intervals such as onset‐to‐imaging and imaging‐to‐specialist‐care as quality indicators for stroke system performance.
4. International organizations and global health funders should incorporate stroke system strengthening, including neurosurgical workforce development and imaging infrastructure, into LMIC health systems investment frameworks.
5. Medical ethics curricula in Ghanaian medical schools and postgraduate training programmes should include case‐based instruction on distributive justice and the ethics of triage in resource‐constrained environments

## Author Contributions

**Kwasi Asare Boadi:** conceptualization, data curation, formal analysis, writing – original draft, writing – review and editing. **Dennis Awedam Achio:** formal analysis, writing – original draft. **Eunice Wilberforce A. Achio:** writing – review and editing.

## Funding

The authors have nothing to report.

## Ethics Statement

The authors have nothing to report.

## Consent

The authors certify that written informed consent was obtained from the patient's next of kin for publication of this report.

## Conflicts of Interest

The authors declare no conflicts of interest.

## Data Availability

The authors have nothing to report.
